# Electron Density Analysis of SARS-CoV-2 RNA-Dependent RNA Polymerase Complexes

**DOI:** 10.3390/molecules26133960

**Published:** 2021-06-28

**Authors:** Nadezhda Palko, Maria Grishina, Vladimir Potemkin

**Affiliations:** Laboratory of Computational Modeling of Drugs, Higher Medical and Biological School, South Ural State University, 454080 Chelyabinsk, Russia

**Keywords:** SARS-CoV-2, RNA-dependent RNA polymerase, electron density analysis, AlteQ, complementarity

## Abstract

The work is devoted to the study of the complementarity of the electronic structures of the ligands and SARS-CoV-2 RNA-dependent RNA polymerase. The research methodology was based on determining of 3D maps of electron densities of complexes using an original quantum free-orbital AlteQ approach. We observed a positive relationship between the parameters of the electronic structure of the enzyme and ligands. A complementarity factor of the enzyme-ligand complexes has been proposed. The console applications of the AlteQ complementarity assessment for Windows and Linux (alteq_map_enzyme_ligand_4_win.exe and alteq_map_enzyme_ligand_4_linux) are available for free at the ChemoSophia webpage.

## 1. Introduction

RNA-dependent RNA polymerase (RdRp) is an essential enzyme for the life cycle of RNA viruses [1] and a promising drug target for many viruses, including SARS-CoV-2 [2]. Today, the development of safe and effective inhibitors of SARS-CoV-2 RdRp is an important problem. Such compounds should have increased selectivity, while the number of side effects with respect to human host proteins should be minimal. Several studies suggest that drugs targeting RdRp could inhibit SARS-CoV-2 [3,4,5]. Compounds capable of tightly interacting with the active site of the RdRp are good candidates for research. Strong binding affinity of molecules to RdRp SARS-CoV-2 leads to the impossibility of performing its function and, consequently, to the destruction of the virus [6,7].

Numerous studies have shown that RNA polymerase Inhibitor favipiravir is a promising drug for COVID-19 [8,9]. The antiviral drug favipiravir is a guanidine analogue targeting RdRp viruses [10,11,12]. Favipiravir is a prodrug. The active form of favipiravir is achieved by phosphoribosylation resulting in the formation of favipiravir-ribofuranosyl-5′-triphosphate (favipiravir-RTP).

The active form is recognized by RdRp as a purine nucleotide that inhibits the activity of the RdRp enzyme and blocks the synthesis of viral RNA [7]. Recently, Naydenova et al. described the structure of favipiravir ribonucleoside triphosphate (favipiravir-RTP) in complex with the RNA-dependent RNA polymerase SARS-CoV-2. The authors determined that the inhibitor is weakly incorporated into the RNA primer strand and exhibits an unusual, nonproductive binding mode at the catalytic site [8]. At the same time, Peng et al. show another productive binding mode of favipiravir-RTP at the catalytic site of SARS-CoV-2 RdRp [9]. Therefore, several variants of binding to the receptor are proposed for one ligand.

The most important stage in drug action is its interaction with its target. The orientation of the drug in the receptor pocket depends on hydrogen bonds, specific short contacts, and van der Waals interactions with the receptor. All these interactions determine the molecular field, which should ensure complementarity [13] of ligands to receptors. The analysis of the electron density distribution allows the identification of non-covalent interactions between the ligand and the receptor [14]. The distribution of the electron density of the outer shells at the ligand-receptor interface is the most important for the formation of the molecular field of covalent bonds and intermolecular contacts.

Niels Bohr formulated the principle of complementarity in 1927 for quantum objects [15]. The concept of complementarity is well-known in physics as the Heisenberg principle [16,17,18]. In biology and chemistry [19], the principle of complementarity is based on supramolecular interactions, which in turn, are based on the geometric, topological, charge correspondence of molecules or their fragments. A large number of works are devoted to the study of the complementarity of interacting structures. Some of the methods are based on Bader’s quantum theory of atoms in molecules (AIM) [20,21,22], while others propose a method for approximating the molecular surfaces of a cavity by inflating a triangular mesh [23,24]. In this article, we propose an alternative method for assessing complementarity based on the AlteQ approach [25,26]. The AlteQ approach with good quality describes the experimental electron density which was determined using low temperature high resolution X-ray diffraction.

In this paper, we studied the electron density of SARS-CoV-2 RNA-dependent RNA polymerase complexes. The aim of the study was to observe the features of the electronic structure of the complexes, registered experimentally. On the one hand, this work is of fundamental importance—understanding how the electronic structures of the enzyme and ligand should be interconnected to ensure complexation. On the other hand, in the long term, the results of the work can be used to determine the degree of complementarity of new ligands to the presented enzyme.

## 2. Materials and Methods

### 2.1. Considered SARS-CoV-2 RNA-Dependent RNA Polymerase Complexes

**Figure 1:**
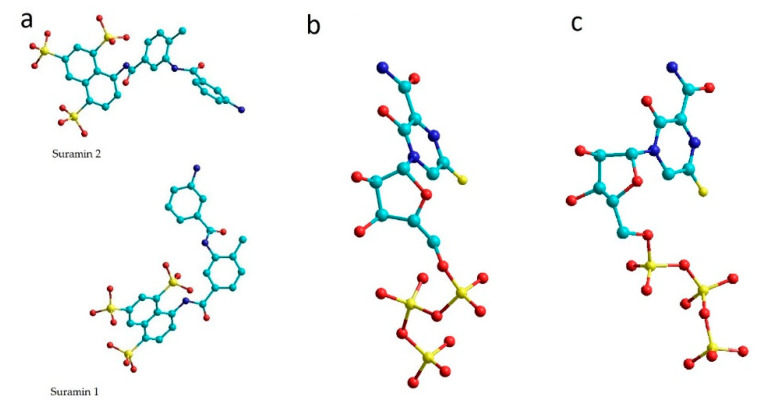
Ligands of 7D4F (**a**), 7CTT (**b**) and 7AAP (**c**) complexes. (colors show: light green—C, yellow—S, red—O, blue—N).

SARS-CoV-2 RNA-dependent RNA polymerase complexes were taken from the Protein Data Bank [27]. The complexes were not changed and were taken for computations as they are. RdRp complexes with favipiravir-RTP (7AAP und 7CTT) and with suramin (7D4F) [28] were selected and analyzed (Figure 1). In 7AAP and 7CTT complexes, the favipiravir-RTP ligand has different conformations.

For all complexes, electron density analysis was performed. The calculations were carried out without taking into account hydrogens.

### 2.2. Calculation of 3D Maps of Electron Density

The calculation of electron density was performed using the improved version of AlteQ method published in [26] in details. AlteQ is an original quantum free-orbital approach, that implements the following principles:

The electron density of molecule is calculated as the sum of atomic contributions at an *m*th point of molecular space with coordinates x_m_, y_m_, z_m_ (Equation (1)),

(1) $$p(x_{m},\;y_{m},\;z_{m})=\sum_{A=1}^{N}p_{A}$$

where *N* is the number of atoms in a molecule, *p_A_* is the A atomic increment in molecular electron density.

The contribution of the electron density of atom A to the molecular electron density is represented as a sum of exponential functions (Equation (2)),

(2) $$p_{A\;}=\sum_{i=1}^{n_{A}}a_{A_{isp}}\exp\left(-b_{A_{i}sp}\cdot R_{A}\right)+\sum_{i=3}^{n_{A}-1}a_{A_{id}}\exp\left(-b_{A_{i}d}\cdot R_{A}\right)+\;\sum_{i=4}^{n_{A}-2}a_{A_{if}}\exp{\left(-b_{A_{i}f}\cdot R_{A}\right)}^{}$$

where *a_Aisp_, b_Aisp_, a _Aid_,b_Aid_, a_Aif_* and *b_Aif_* are AlteQ atomic parameters describing the *i*-th *sp*-orbital, *d*-orbital and the *f*-orbital of *A* atom, *n_A_* is the period number of the *A* atom, *R_A_* is the distance between the *A* atomic center and the *m*th point.

For the outer shells, the electron density can be calculated as follows (Equation (3)).

(3) $$p_{A\left(outer\right)\;}=a_{A_{nsp}}\exp\left(-b_{nsp}\cdot R_{A}\right)+a_{A{\left(n-1\right)}_{d}}\exp\left(-b_{A{\left(n-1\right)}_{d}}\cdot R_{A}\right)\;+a_{A{\left(n-2\right)}_{f}}\exp{\left(-b_{A{\left(n-2\right)}_{f}}\cdot R_{A}\right)}^{}$$

The value of electron density of inner shells ($p_{A\left(inner\right)}$) (Equation (4)) has been estimated as follows:

(4) $$p_{A\left(inner\right)}=p_{A\;}-p_{A\left(outer\right)\;}$$

The function *p_A_* is based on analysis of the relationship between the logarithm of the experimental electron density derived from the high resolution X-ray diffraction experiment and the distance to the nuclei. AlteQ makes it possible to reconstruct the distribution of electron density in a short time and gaining results, which are very close to the experimental ones. The AlteQ method is especially valuable for studying electron density in large molecular structures, where the electron density analysis is a very time-consuming procedure. The applicability of AlteQ approach for studying large molecular structures has been shown in previous works. AlteQ was used to study the electronic properties of the structures of enzyme—ligand complexes [25,26,29], to study the orders of bonds [30], and photovoltaic properties of dye—TiO_2_ nanoparticles complexes [31,32].

Since outer shells play the most important role in the enzyme-ligand interactions, we desided to estimate the contribution of the electron density of the outer shell of the enzyme ($\rho_{E}$, see Equation (5)) and the ligand ($\rho_{L}$, see Equation (6)) to the *m*th point as follows,

(5) $$\rho_{E}\left(x_{m},y_{m},z_{m}\right)=\overset{N_{enzyme}}{\underset{\begin{array}{c} A=1\\ A\in enzyme \end{array}}{\sum }}\rho_{Am\left(outer\right)}\;$$

(6) $$\rho_{L}\left(x_{m},y_{m},z_{m}\right)=\overset{N_{ligand}}{\underset{\begin{array}{c} A=1\\ A\in ligand \end{array}}{\sum }}\rho_{Am\left(outer\right)}\;$$

where $N_{enzyme}$ and $N_{ligand}$ are the numbers of atoms of the ligand and the enzyme.

Intermolecular interactions in the ligand—RdRp system determine the molecular field, which in turn, should ensure the complementarity of the ligand to the receptor. The molecular field is sensitive to changes in intermolecular interactions, caused by ligand movement in the process of interaction with the RdRp. An important parameter characterizing intermolecular interactions is the electron density. The electron density in the region of intermolecular interaction can be used to assess the enzyme-ligand complementarity. In this regard, we calculated 3D maps of the electron density for all complexes. In this case, points with an electron density of the receptor and ligand of more than 0.001 au. (0.001 e/Bohr^3^) were considered. This limitation characterizes the boundary of the atom in Bader’s theory. The electron density was calculated in the cubic grid with the distance between nearest junctions 0.1 Å (each junction is the *m*th point) [29,33,34].

### 2.3. Complementarity Factor

For each complex, we observed an empirical dependence described by Equation (7),

(7) $$fc=a-b\cdot sumR_{L}R_{E}$$

where *a* and *b* are parameters of the equation.

The parameter fc (Equation (7)) is called complementarity factor. It most effectively describes the relationship of electron densities contributions of the ligand and the receptor at the *m*th point of the molecular space, was computed. The complementarity factor is expressed through the sum of two contributions provided by the receptor and the ligand (Equation (8)),

(8) $$fc=\ln\left(\rho_{E}\cdot \frac{\rho_{EC}}{N_{E}}\right)+\ln\left(\rho_{L}\cdot \frac{\rho_{LC}}{N_{L}}\right)$$

where $\rho_{LC}$ is the electron density of the outer shell in the center of the highest-contributing ligand atom, $\rho_{EC}$ is the electron density of the outer shell in the center of the highest-contributing receptor atom, $N_{L}$ is the atomic number of the highest-contributing ligand atom, and $N_{E}$ is the atomic number of the highest-contributing receptor atom.

The sumR_L_R_E_ parameter is the sum of the distances between the *m*th point and the ligand atom having the highest contribution to $\rho_{L}$ at this point (R_L_), and the distance between the *m*th point and the receptor atom having the highest contribution to $\rho_{E}$ at this point (R_E_) (Equation (9)):

(9) $$sumR_{L}R_{E}=R_{L}+R_{E}$$

We have implemented the complementarity assessment in the alteq_map_enzyme_ligand_4 program for Windows and Linux, now the console applications of the proposed method are publicly available at www.chemosophia.com, accessed on 28 June 2021.

Based on the Equations (7)–(9), we can express the AlteQ complementarity principle as follows (Equation (10)):

(10) $$\left\{\left(\frac{\rho_{L}\;\rho_{LC}}{N_{L}}\right)exp\left(b\;R_{L}\right)\right\}\left\{\left(\frac{\rho_{E}\;\rho_{EC}}{N_{E}}\right)exp\left(b\;R_{E}\right)\right\}=\exp\left(a\right)$$

We can designate the ligand and the enzyme contributors as $\sigma_{E}$ (Equation (11)), and $\sigma_{L}$ (Equation (12)), respectively:

(11) $$\sigma_{E}=\left(\frac{\rho_{E}\;\rho_{EC}}{N_{E}}\right)exp\left(b\;R_{E}\right)$$

(12) $$\sigma_{L}=\left(\frac{\rho_{L}\;\rho_{LC}}{N_{L}}\right)exp\left(b\;R_{L}\right)$$

If $R_{L}$ and $R_{E}$ are measured in Å, $\sigma_{E}$ and $\sigma_{L}$ are measured in e/Å^6^.

Then we can get a formula that closely resembles Heisenberg’s principle for complementary properties in physics (Equation (13)):

(13) $$\sigma_{E}\sigma_{L}=\exp\left(a\right)$$

Based on *a*- and *b*-parameters determined for the experimental complexes, we can reconstruct the pattern of the desired electronic structure of the new promising drug. The pattern can be represented as follows (Equation (14)):

(14) $$\sigma_{L}=\exp\left(a\right)/\sigma_{E}$$

Further, the actual values $\sigma_{L\left(new\right)}$ of a new molecule can be compared with the desired ones $\sigma_{L}$ to ensure maximum similarity. In the case of minimum difference, it is possible to establish the most complementary position of the ligand in the RdRp site.

### 2.4. Complementarity Assessment

**Figure 2:**
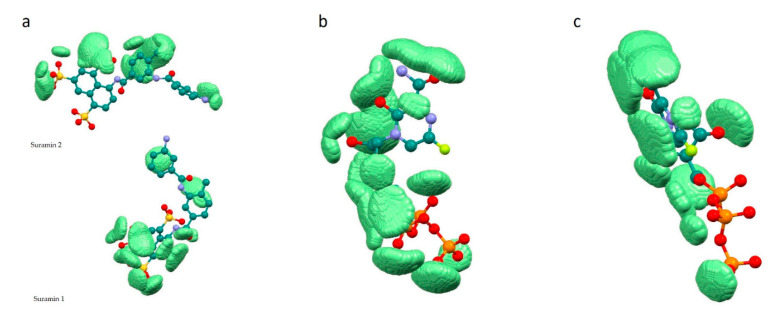
Complementary field (light green) of ligand—receptor complexes. ($\rho_{L}>0.001$ a.u. and $\rho_{E}>0.001$ a.u.): (**a**)—7D4F, (**b**)—7CTT, (**c**)—7AAP.

Dependencies (Equation (7)), *a* and *b* parameters and statistical characteristics (correlation coefficient R, standard error of estimate, standard errors of the *a* and *b* parameters, Fisher’s criterion) have been found using the method of statistical analysis—Multiple Regression Model. Graphs of the dependencies *fc = f(sumR_L_R_E_)* were built using Statistica 13 software [35]. Coordinates of points *m* of the complementary field with $\rho_{L}>0.001$ a.u. and $\rho_{E}>0.001$ a.u. and corresponding to the significant overlap of the ligand with the receptor pocket have been printed in pdb files of complexes using our own Windows application uni_coord_one_mol_pdb.exe. The result of visualization using Mercury 3.9 [36] is shown in Figure 2.

## 3. Results and Discussion

Figure 2 demonstrates the localization of zones (the set of considered points *m* with $\rho_{L}>$ 0.001 a.u. and $\rho_{E}>0.001$ a.u.) in which there is significant overlap of the ligand with the receptor pocket, near the ligands. In other words, these are zones of space with a receptor and ligand electron density of more than 0.001 au. (0.001 e/Bohr^3^). In this regard, these zones determine the complementary field of the ligand and receptor in terms of electron density.

**Figure 3:**
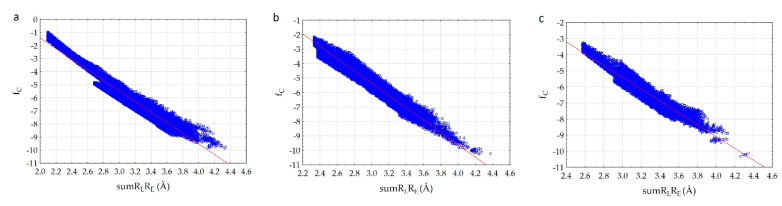
Correlation of the complementarity factor (fc) and the sum of the distances (sumR_L_R_E_): (**a**)—7D4F (suramin complex), (**b**)—7CTT (favipiravir-RTP complex), (**c**)—7AAP (favipiravir-RTP complex).

We have established that fc correlates with the sumR_L_R_E_ parameter (Figure 3) very well.

**Figure 4:**
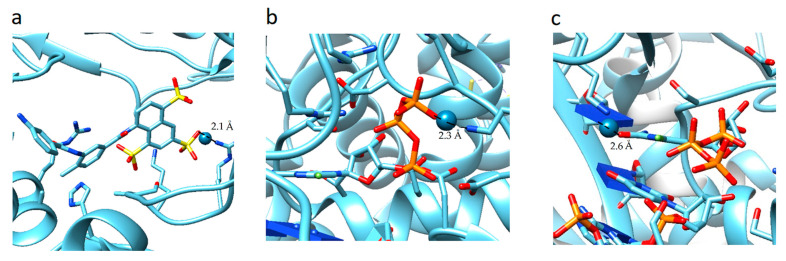
Location of point corresponding to the maximum complementarity factor (fc) for complexes: 7D4F, 7CTT, 7AAP: (**a**) 7D4F, 2.1 Å is the distance between the sulfo group of suramin and the guanidine component of arginine, (**b**) 7CTT, 2.3 Å distance between protease lysine and triphosphate group of favipiravir-RTP, (**c**) 7AAP, 2.6 Å distance between RNA cytosine and oxygen of the amide group of favipiravir-RTP.

The analysis of the results showed that the character of the dependence of fc on the sumR_L_R_E_ value for all complexes practically does not change (Figure 3). In all cases, with the decrease of the sum of distances (sumR_L_R_E_), the overlap of the electron clouds of the ligand and the receptor is enhanced and fc increases. The increase in the factor is associated with a change in the nature of interactions in the system from less effective van der Waals, to more effective specific contacts and hydrogen bonds. The maximum allowable value of the complementarity factor should correspond to the contact distances at which, according to the Pauli principle, there is no overlap of the inner electron shells. Therefore, the minimum value of the sumR_L_R_E_ should also correspond to the absence of overlapping of the inner electron shells. Therefore, the minimal values of sumR_L_R_E_ and maximal values of fc were investigated for all complexes. We found that in all cases, these values ensure that the Pauli principle is met. All complexes are characterized by the absence of overlapping of the inner electron shells; the value of electron density of inner shells ($p_{A\left(inner\right)}$) does not exceed 10^−8^ a.u. in the space of the receptor-ligand overlaps ($\rho_{L}>$ 0.001 a.u. and $\rho_{E}>0.001$ a.u.). The minimal values of sumR_L_R_E_ and maximal values of fc are different for all complexes. This indicates different types of interactions in the complexes (Figure 4). The 7D4F complex consists of RdRp and two suramin molecules. Intermolecular interaction corresponding to the minimum distance of 2.1 Å, is observed only for the second suramin molecule. This is hydrogen bond of one out of three sulfo groups (position 3) of suramin with the guanidine moiety of arginine (Figure 4a). In the 7CTT and 7AAP complexes, the favipiravir-RTP ligand has different conformations and is located differently in the RdRp cavity. In this regard, the interactions between the ligand and the receptor corresponding to the minimum distance are different. In the 7CTT complex, the distance of 2.3 Å corresponds to the interaction between the protease lysine and the favipiravir-RTP triphosphate group (Figure 4b), and in 7AAP, the minimum distance 2.6 Å corresponds to the interaction of the RNA cytosine with the oxygen of the amide group of favipiravir-RTP (Figure 4c). These contacts can be attributed to the most effective interactions that characterize the mechanism of the interactions.

The values of the a-and b-parameters (Equation (7)) determined for the considered complexes, Standard Error for a-and b-parameters (St. Err.), Standard Errors of estimate, Fisher’s criterion (F-test), amount of *m* points and the correlation coefficient R are presented in Table 1. This information can be used for docking new compounds and select the most promising antiviral agents, which have the most similar electronic structure to the known ligands.

An analysis of the results showed that the a- and b-parameters are different for all complexes and depend on the atoms involved in intermolecular interactions. The correlation coefficients of the dependencies are 0.966–0.973. Therefore, we can presuppose that the whole proposed principle of complementarity lies precisely in the fact that from the entire set of atoms of the enzyme and the ligand, only a pair of the enzyme and the ligand atoms which make the most significant contribution to the electron density at the *m*th point can be isolated. This provides a high correlation of the complementarity factor and sumR_L_R_E_ value. In the case when two or more atoms make a significant contribution to the *m*th point, the dependence should worsen, and the correlation coefficient should decrease. Therefore, in the considered complexes, the interactions between the enzyme and the ligand are carried out in such a way that the pair atom-atom interactions are mainly observed and the number of bifurcate intermolecular bonds is minimized. Based on the correlation coefficients, it can be concluded that the 7CTT complex is characterized by a slightly higher complementarity of RdRp and ligand compared to the 7D4F and 7AAP complexes. This model provides the monotonic increase in the electron density overlap with decreasing intermolecular distance, taking into account the entire spectrum of intermolecular interactions from weak van der Waals to strong hydrogen bonds. Indeed, a comparison of the graphs of dependences for various complexes showed that for the 7CTT complex the dependence is more uniform. The graph width is highly dependent on the variety of ligand and receptor contacts. The 7D4F complex is characterized by less diverse contacts and the ligand of 7D4F complex interacts with a smaller number of the receptor atoms than ligands of 7CTT and 7AAp complexes.

**Figure 5:**
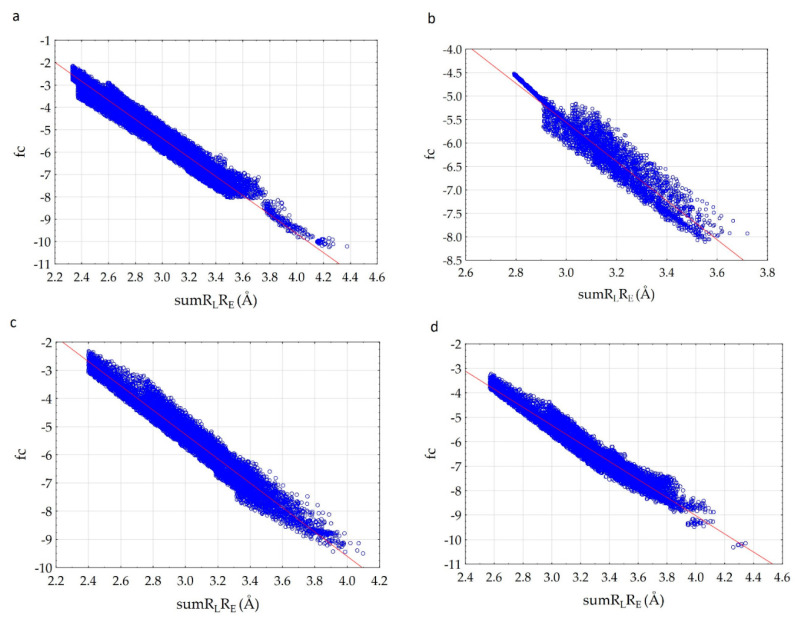
Correlation of complementarity factor (fc) with the sum of the distances (sumR_L_R_E_): 7CTT enzyme—favipiravir-RTP (**a**), 7AAP enzyme—favipiravir-RTP (**b**), 7CTT RNA—favipiravir-RTP (**c**), 7AAP RNA—favipiravir-RTP (**d**).

Complexes 7CTT and 7AAP have the same ligand—favipiravir-RTP, but the graphs of dependences differ. We decided to determine the reason for the found difference. For this, we analyzed the structure of the 7CTT and 7AAP complexes. The molecular field of the ligand is complementary to the molecular field of the receptor. In the 7CTT and 7AAP complexes, the favipiravir-RTP is surrounded by RNA and enzyme. Each subunit has its own molecular field. Then, the molecular field of the receptor will be defined as a superposition of the subunit fields, and the ligand field should be complementary to both RNA and enzyme fields. To determine the most effective binding of the molecule to the receptor, a comparative analysis of the relationship between the complementarity factor and sumR_L_R_E_ in the ligand complexes with each RdRp subunit was carried out; “ligand—RNA” and “ligand—enzyme” interactions were considered separately. Analysis was performed for both 7CTT and 7AAP complexes. As a result, it was found that in all cases there is a linear increase of fc with a decrease of sumR_L_R_E_. In this case, the plots for the 7CTT and 7AAP structures differ quite strongly (Figure 5). This feature indicates a strong effect of the conformational state of the ligand on the complementarity.

**Figure 6:**
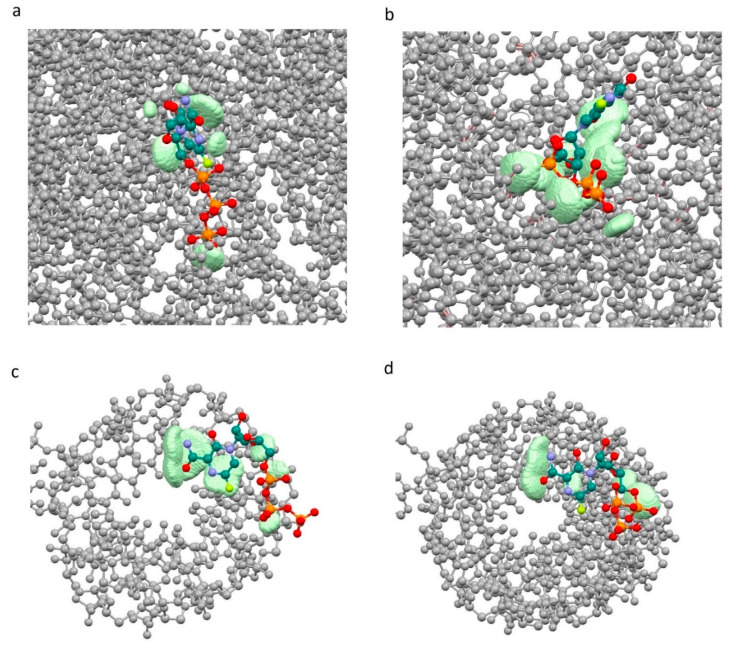
Complementary field of complexes 7CTT and 7AAP: 7AAP enzyme—favipiravir-RTP (**a**), 7CTT enzyme—favipiravir-RTP (**b**), 7AAP RNA—favipiravir-RTP (**c**), 7CTT RNA—favipiravir-RTP (**d**) (grey—enzyme (**a**,**b**) and RNA (**c**,**d**), light green—complementary field).

The shape of the graph *fc = f(sumR_L_R_E_)* for “ligand-enzyme” interactions is more uniform for the 7CTT complex as compared to the 7AAP complex. For 7AAP complex, a narrow band is observed on the graph for sumR_L_R_E_ 2.8–3.0 Å, and after reaching 3.0 Å, the narrow band becomes wide and loose. This kind of uneven change in the fc indicates that for different sumR_L_R_E_, different numbers of atoms are involved in the intermolecular interactions. The complementary fields of 7CTT enzyme and 7AAP enzyme with favipiravir-RTP are shown in Figure 6a,b. In contrast to the enzyme—ligand interactions of the 7CTT and 7AAP complexes, the RNA—ligand interactions of these complexes are more similar. In this case, a monotonic increase in the descriptor with a decrease in sumR_L_R_E_ is observed. This indicates a uniform overlap of the electron clouds of the ligand with the RNA at each *m*th point. Thus, the favipiravir-RTP conformation observed in the 7CTT complex is complementary to both RNA and the enzyme (Figure 6). In the case of the favipiravir-RTP (7AAP) conformation, it can be noted that the ligand interacts more efficiently with RNA than with the enzyme.

Correlation coefficients R1, as well as c- and d-parameters of the equation *fc = f(sumR_L_R_E_)* for “enzyme—ligand” and “RNA—ligand” interactions of 7CTT and 7AAP complexes are shown in Table 2.

The results presented in the Table 2 confirm the stronger complementarity of the “ligand—RdRp” in the 7CTT structure than in the 7AAP complex. The correlation coefficient R1, as well as c- and d-parameters obtained for the structures 7CTT and 7AAP show that the maximum complementarity of the ligand to the enzyme is achieved only if the ligand is complementary to all structural subunits of the receptor, namely RNA and the enzyme. The results obtained can be used to establish the reliability of docking of new structures.

## 4. Conclusions

In this article, a study of complementarity in the SARS-CoV-2 RNA-dependent RNA polymerase complexes with ligands was carried out. The complementarity assessment was based on the overlap of the electron clouds of the ligand and the receptor. The electron density was calculated using the original quantum free-orbital AlteQ approach. We have determined the complementarity factor relating to electron density contributions of the ligand and the receptor to each other. The relationship between the complementarity factor and the sum of the distances between contacting atomic centers has been established. It was found that the maximum complementarity of the ligand to the RdRp is achieved in the case of the complementarity of the ligand to both RNA and enzyme subunits of the receptor. The results can be used to predict biological activity, molecular docking, as well as to study the mechanism of drug action. Equations describing complementarity can be used to assess the correct binding pose of a new ligand to the receptor by comparing a- and b-parameters of the equation, correlation coefficient or standard error of estimate of modeled complexes with the data found in the current work.

## Figures and Tables

**Table 1:** Values of the a- and b-parameters, Standard Error for a-and b-parameters (St. Err.), Standard Errors of estimate, Fisher’s criterion (F-test), total points and the correlation coefficient R.

Complexes	a	St. Err. a	b	St. Err. b	St. Err. of Estimate	F-Test	Amount of *m* Points	R
7D4F	6.683	0.017	4.0547	0.0052	0.35	600340	33838	0.973
7CTT	7.406	0.018	4.2539	0.0059	0.30	522541	25622	0.976
7AAP	5.632	0.029	3.6824	0.0089	0.30	169352	12149	0.966

**Table 2:** Values of the c- and d-parameters, Standard Error for c- and d-parameters (St. Err.), Standard Errors of estimate, Fisher’s criterion (F-test), amount of *m* points and the correlation coefficient R1 for complexes 7CTT and 7AAP.

Complexes	c	St. Err. c	d	St. Err. d	St. Err. of Estimate	F	Amount of *m* Points	R1
7CTT enzyme—L	7.365	0.021	4.2531	0.0070	0.30	373456	17060	0.978
7CTT RNA—L	7.697	0.033	4.326	0.011	0.32	165549	8481	0.975
7AAP enzyme—L	6.954	0.074	4.172	0.024	0.24	31314	3286	0.951
7AAP RNA—L	5.755	0.028	3.6953	0.0086	0.27	185132	8829	0.977

## Data Availability

Publicly available datasets were analyzed in this study. This data can be found here: www.chemosophia.com.
